# Annual Meeting of the American Medical Association

**Published:** 1866-06

**Authors:** 


### Miscellaneous.

### ANNUAL MEETING OF THE AMERICAN MEDICAL ASSOCIATION.

### First Day.

This body met May 1st, at 11 o'clock, in the main hall of the Concordia Opera House, on Eutaw street, near German street, Baltimore, there being about two hundred and fifty delegates present. The President, Dr. D. H. Storer, of Boston, took the chair. After being called to order, a very impressive prayer was offered by Rev. Dr. Spees.

The Chair stated that the first business under the rules was the reception of communications from the Committee of Arrangements. Dr. C. C. Cox, of that Committee, in an eloquent address, gave a warm welcome to the Association, and hoped that when the short stay of the members is ended, they will have cause to retain kindly remembrances of the Monumental City. He expressed his regret that so few delegates from the South were present, and hoped now that peace has come they will again return and aid the Association with their learning and experience in the great work the profession has before it. He paid a high compliment to the surgeons on both sides during the late war, and referred in pathetic terms to the many learned men who have been taken away by death since the Association met in Baltimore eighteen years ago. He closed by again warmly welcoming the visiting brethren.

The hour of meeting during the session was fixed at 9 A. M.

Under a suspension of the rules, Dr. C. C. Cox presented several papers in reference to the case of Dr. Montrose A. Pallen, of St. Louis, whose name was last year erased from the list of delegates, on charges preferred against him, showing clearly the innocence of Dr. P., and moved that they be referred to the Committee on Medical Ethics.

Dr. Toner, of Washington, moved, as an amendment, that the Association take up the subject without any reference to the Committee.

Dr. Ordway, of Boston, advocated the amendment, and stated that as the action of the previous year had been done without reference to a committee, he thought that the reparations should be as decided and prompt in the present instance. Dr. O. further remarked that as one of the protestants to the action of the Association last year, it was eminently proper that this apology should be made by a direct vote.

Dr. Davis, of Illinois, supported the original motion, on the ground that, as the action of the Association in this matter was wide spread upon the records, it was only just to Dr. Pallen that his vindication of the charges should be as full and thorough on the journal as possible. By a mere motion to rescind this would not be done, and through a committee a full report and thorough exoneration would be made public.

Dr. Cox stated that he had an interview with Dr. Pallen on Monday, and that Dr. P. preferred that the matter should be referred.

Dr. Owens, of Maryland, asked for information with regard to the case, whereupon the Chair stated that at the session held last year in Boston, charges had been preferred against Dr. Pallen, then a member of the Association, of disloyal conduct and practices, and a resolution had been there passed to erase his name from the list of members, and the question now was to restore Dr. Pallen to his membership, the statements against him having been proved to be unfounded.

The main question having been called, the papers were referred to Drs. Hooker, Brinsmade and Davis, as a Committee on Medical Ethics.

A list of names of delegates, from the Committee on Credentials, was read by the Secretary.

Under the rule for the reception of members by invitation, James E. Rives, of Fairmount, West Virginia, was received.

Dr. Thomas E. Bond moved that the committee to whom was referred the case of Dr. Pallen, be instructed to report the expression of their profound regret at the Association being hurried into its unjust action to Dr. Pallen, and that they hope that Dr. Pallen will accept this acknowledgment as an expression of a frank apology for the great wrong done him; which, together with an amendment, was laid on the table.

Dr. Storer, President of the Association, read his annual address, which was listened to attentively, and, on motion, was referred to the Committee on Publication. A unanimous vote of thanks was tendered to Dr. Storer for his excellent address.

The Committee on Ethics, to whom was referred the case of Dr. Pallen, announced their readiness to report, which was read, and speeches made thereon by Drs. Tyler, Bond, Ordway and Owens, the last named moving that the report be re-committed, with instructions to incorporate Dr. Bond's resolution, which had been previously tabled.

Dr. Hooker, from the Committee, then presented the following amended report, with the preamble and resolution, which were unanimously adopted:

"The Committee to whom were referred the papers in relation to the expulsion of Dr. Montrose A. Pallen, at the meeting of the Association in Boston, respectfully report that they have examined the documents and evidence referred to them, embracing papers endorsed by Lieut. Gen. U. S. Grant, the Vice Consul of the United States at Montreal, and many citizens of Missouri, and are fully satisfied that the statements on which his expulsion was based were entirely unfounded, and therefore, regretting the injustice done both to Dr. Pallen and the Association, we recommend the following resolution:

"*Resolved*, That the preamble and resolution adopted by the Association at their annual meeting in Boston, June, 1865, expelling Dr. Pallen, be hereby rescinded, and that Dr. Montrose A. Pallen be restored to his previous membership in the Association."

Dr. Ordway sustained the same, and moved that a committee of three be appointed to meet Dr. Pallen and inform him of the unanimous action of the convention.

Dr. Owens moved as an amendment that Dr. Cox be chairman, which was accepted by Dr. Ordway.

The chair then appointed Drs. Cox, Ordway and Sayre.

The committee soon after returned with Dr. Pallen, who was conducted to the platform amidst applause, and presented to the Association as perfectly exonerated from all charges. Dr. Pallen, in reply, stated that he was deeply gratified at the action of the Association; that its action in regard to him had almost overwhelmed him, but that, whilst buffeting with the waves of infamy and disgrace, he was comforted by the bright light of conscientious performance of duty. The man who would have dared to reproach him, with even a hint of poisoning the Croton reservoir, would have been spurned with contemptuous indignation. On the battlefields of the war he had been stayed by no danger in the performance of his professional duty, and whether the wounded man was clad in grey or in blue, he had ministered to him, regardless of the time or place. He returned his thanks again to the Association, and also to Dr. Cox for his manly conduct, and closed with the expression of his gratitude for the warmth of his reception.

The call of special committees and volunteer papers on professional subjects for the purpose of being referred to appropriate sections was then made, lasting some time.

Dr. Jewell announced the presence of Dr. Marsden, from Canada, and moved that he be received as a permanent member, and be invited to take a seat on the platform; which was agreed to.

On motion, Dr. E. Brown-Séquard, of Boston, was invited to address the Association to-day, at 11 A. M., on the treatment of nervous disorders.

After some business announcements, the Association adjourned until 9 A. M., the various sections meeting during the afternoon to prepare business.

In the evening there was a promenade concert and entertainment to the visiting delegations at Concordia Hall, given under the auspices of the committee of arrangements, which was, notwithstanding the rain, fully attended, many ladies being present. The next evening private soirees were to be given the members of the Association by Dr. C. C. Cox, No. 23 McCulloh street, Dr. T. S. Bond, 82 Read street, and Surgeon Jos. Simpson, U. S. A., No. 40 Calhoun street. The entertainment tendered by the Mayor and City Council will follow.

SECOND DAY, MAY 2D.

The Association was called to order by the President, Dr. D. H. Storer, at 9 A. M.

The Committee on Epidemics, Meteorology, &c., having been called upon, Dr. Davis stated that Dr. Hamill, of Indiana, had presented a report, which he had taken to the Section on Epidemics, &c.

Dr. Cox made an additional report from the Committee on Arrangements on Railroads, that invitations had been received from Drs. Smith and Donelson, for the members to visit their houses that evening. He also recommended the following gentlemen as members by invitation: Drs. Jno. A. Reed, W. Whitridge, L. M. Eastman, of Baltimore; Peter Parker, of China. They were elected.

On motion of Dr. Davis, the order of business was suspended.

The report of the Committee on Publication was read and accepted.

On motion of Dr. Sayre, of New York, the Publication Committee were authorized to enforce strictly the rules in regard to proofs, &c.

The Treasurer then read his report, which was referred to the Committee on Publication.

On motion, the order of business was resumed.

On motion of Dr. Davis, a recess of fifteen minutes was taken by the Association, to allow of the appointment of members of the Nominating Committee.

*The Nominating Committee.*—On the resumption of business, the following members of that Committee were announced:—J. C. Weston, Me.; J. C. Eastman, N. H.; Wm. McCollom, Vt.; J. R. Bronson, Mass.; D. King, R. I.; W. Woodruff, Conn.; J. C. Hutchison, N. Y.; W. Pierson, Jr., N. J.; H. F. Askew, Del.; John L. Atlee, Pa.; J. J. Cockrill, Md.; M. A. Pallen, Mo.; N. S. Davis, Ill.; W. Lockhart, Ind.; J. M. Witherwax, Iowa; N. R. Bozeman, Ala.; C. M. Stockwell, Mich.; H. Van Dusen, Wis.; T. A. Atchison, Tenn.; G. Fries, Ohio; G. Tyler, D. C.; W. M. Charters, Geo.; Josiah Simpson, U. S. A.; Ninian Pinkney, U. S. N.; Greensville Dowell, Texas.

Dr. W. Hooker offered the following resolution, which was unanimously adopted:

"*Resolved*, That no report or other paper shall be presented to this Association unless it is so prepared that it can be put at once into the hands of the Secretary, to be transmitted to the Committee on Publication."

Dr. Wister, of Pa., offered the following, which was adopted:

"*Resolved*, That Drs. Grafton Tyler, W. P. Johnston, and Jas. M. Toner, of D. C., be a Committee to procure a room in the Smithsonian Institution for the preservation of the Archives of the Association."

The Committee on Medical Education not having prepared a report, Dr. J. F. Hibberd offered instead thereof the following preamble and resolution, and moved that it be adopted as the sentiment of the Association:

"*Whereas*, Two-thirds of the Medical Colleges of the States of Ohio, Michigan, Illinois, Iowa, Missouri, Kentucky and Tennessee, by delegates in convention assembled in Cincinnati, on the 24th of April ult., did, by resolution, unanimously adopted, declare their willingness to make their annual college sessions to continue for six months, and to establish a uniform rate of fees, if the other principal colleges of the country will coöperate; now, therefore,

"*Resolved*, That the American Medical Association hereby expresses its warmest approbation of the action of the above recited colleges, and expresses the hope that every medical college in the Union will concur in the proposition thus made."

On motion of Dr. Taylor, of Iowa, its consideration was postponed till 11 A. M., on Thursday, to be acted upon in Committee of the Whole.

Dr. C. A. Lee, of New York, commenced reading his report upon Medical Literature. He divided up his subject as follows:— I. Periodical Medical Press. II. Medical Literature of the War. III. Literature of the Sanitary Commission and Sanitary Sciences. IV. State and County Society Transactions. V. Literature of Special Subjects and Specialties. VI. Literature of Pharmacy and Materia Medica. VII. Of Vital Statistics. VIII. Of Life Assurances. IX. And of Introductory Lectures.

He was interrupted at 11 for the regular order of business, which was the lecture of Dr. Brown-Séquard, on the Treatment of Functional and Organic Diseases of the Nerves.

On motion of Dr. Raphael, of N. Y., the thanks of the Association were tendered to Dr. Brown-Séquard for his interesting, able and eminently practical lecture, and he was requested to furnish an abstract for publication.

Dr. C. A. Lee then resumed the reading of his report.

After this had continued for some time, on motion of Dr. Toner, the further reading was discontinued, and the paper referred to the Committee on Publication.

Dr. Samuel D. Gross, Chairman of the Committee on Medical Education, reported that he had not prepared a report, and asked that the Committee be discharged, which was granted.

*Report of Prize Committee.*—Dr. E. Eliot, Secretary of the Committee on Prize Essays, read the report of that Committee.

On breaking the seals, Dr. W. F. Thoms, of New York city, was ascertained to be the author of the "Essay on Health in Cities," &c., and was entitled to the first prize, and Dr. S. R. Percy, of New York, on "Digitaline," &c., to the second.

On motion, the paper on Angular Curvature of the Spine was referred to the Section on Surgery.

The report of the Committee on Medical Ethics having been offered, it was made the special business for 9.30 on Thursday.

Dr. Marsden, of Canada, having been announced as desirous of making some remarks on Cholera, on motion, it was agreed that he should follow immediately after the report on Medical Ethics.

Dr. Cohen offered a paper on Paralysis of the Vocal Chords and Aphonia, &c. Referred to the Section on Surgery.

Dr. H. R. Storer offered a paper on the "Clamp Shield," an instrument designed to lessen the dangers of extirpation of the uterus by abdominal section.

Dr. Bozeman, of Ala., was introduced to the Association, and on motion of Dr. Holton, he was made the member of the Committee on Nominations for Alabama.

Dr. Askew offered the following resolution on the death of Dr. Couper, which was unanimously adopted:

"*Whereas*, We have heard with profound regret of the death of our deservedly esteemed friend and associate, James Couper, M. D., of Delaware, late Vice President, and one of the founders of the American Medical Association; and whereas, we desire to express our high appreciation of his worth as a man, and valuable and untiring energy in the cause of medical science; mild, modest and unassuming, of devoted piety, he was firm, constant and reliable; a strict adherent to the ethics of the profession, he occupied a front rank, and died beloved, respected and lamented by all who knew him.

"*Resolved*, That in the death of Dr. James Couper we have lost a friend and brother, and that we sincerely and deeply condole with his sorrow-stricken widow and family, and that the Secretary be authorized to forward a certified copy of these resolutions to his family."

Dr. Toner, of D. C., offered the following resolution, which was adopted:

"*Resolved*, That instead of yearly reprinting the list of members of the American Medical Association with the Transactions of the same, the Secretary be instructed to prepare and have printed in pamphlet form, a triennial alphabetical catalogue, containing the Constitution of the Association, and a list of members, with their full names, designating their residences, the year of their admission, arrearage of yearly dues, the offices they may have held in this body, and in case of death or resignation, the year, and distribute the same among the contributing members."

On motion, the resolution was referred to the Committee on Publication.

Dr. J. C. Hughes, of Iowa, offered a paper on Lithotomy, which was referred to the Section on Surgery.

Dr. Taylor, of Iowa, introduced a resolution for the appointment by the President of the Association of a member from each State, to memoralize Congress for an appropriation to publish the reports and documents of the Surgeon-General of the United States.

Dr. Pallen recommended that the reports and documents of the like character connected with the rebel army be also referred to the same committee for access to the same. Dr. Pallen, after some discussion, withdrew his amendment.

The original motion was carried.

It was then moved that the President announce said Committee on Thursday morning. The meeting then adjourned.

[TO BE CONTINUED.]

---

The Surgeon-General has had constructed a beautiful model of the Hicks United States Army Hospital at Baltimore, Md., which he designs to send to France, to be exhibited at Paris Exposition of 1867. The model is of wood, and is made on the scale of one inch to twenty feet.

